# Phage vB_KpnS_MK54: lPS-associated factors involved in phage adsorption and depolymerase-driven anti-biofilm effects

**DOI:** 10.3389/fmicb.2026.1963357

**Published:** 2026-10-09

**Authors:** Yan Zeng, Zhaomin Wu, Zhuangkan Yan, Yin Wang, Zexiao Yang, Yixin Huang, Suizhong Cao, Xueping Yao

**Affiliations:** 1 College of Veterinary Medicine, Sichuan Agricultural University, Chengdu, China; 2 Key Laboratory of Agricultural Animal Diseases and Veterinary Public Health of Sichuan Province, Chengdu, China

**Keywords:** antibiotic resistance, biofilm, depolymerase, *Klebsiella pneumoniae*, phage, phage adsorption

## Abstract

Understanding phage *K. pneumoniae*–host interactions is essential for elucidating phage infection mechanisms and developing phage-derived antibacterial strategies. This showed that lipopolysaccharide (LPS) plays an important role in phage vB_KpnS_MK54 adsorption by constructing the isogenic deletion mutants LPKPΔmsbA and LPKPΔlpxB, in which phage adsorption was reduced by 2–3 log compared with the wild-type strain. Biofilms are associated with persistent infections and increased tolerance to antimicrobial treatment, posing a significant clinical challenge. We further characterized the recombinant depolymerase Dep_Zy, which exhibited potent depolymerase and antibiofilm activities, including depolymerase activity at 0.1 μg/mL, 55% inhibition of biofilm formation at 10 μg/mL, and 30% degradation of mature biofilms at 1 μg/mL. Notably, Dep_Zy potentiated the activity of conventional antibiotics, resulting in a 1–3 log reduction in biofilm-associated bacterial counts when combined with kanamycin, gentamicin, or streptomycin. These findings elucidate the molecular basis of phage–host interactions and highlight Dep_Zy as a promising enzymatic strategy for controlling multidrug-resistant *Klebsiella pneumoniae* through biofilm disruption and antibiotic potentiation.

## 1 Introduction

*Klebsiella pneumoniae*, a critical zoonotic pathogen, poses severe clinical resistance due to its escalating an antibiotic resistance, causing pneumonia, urinary tract infections and systemic infections, etc., (Singh et al., 2024; Bruchmann et al., 2021; Sá-Pessoa et al., 2025). Its virulence is attributed to surface components like lipopolysaccharide (LPS) and capsular polysaccharide (CPS), which evade host immune recognition (Jiang et al., 2025; Ramos et al., 2026; Kawooya et al., 2026). LPS composed of O-antigen, lipid A, and core polysaccharides, not only contributes to pathogenicity but also serves as a primary phage receptor (Ibrahim et al., 2025; Mohr et al., 2022). The antibiotic resistance caused by *K. pneumoniae* biofilms has become a popular research focus. These structured microbial communities encased in self-produced extracellular polymeric substances (EPS), exhibit intrinsic resistance to both antibiotics and host immune defenses (Yao et al., 2022; Sahu et al., 2025). While conventional treatments eliminate planktonic bacteria, biofilm-embedded cells persist as reservoirs for recurrent infections, particularly in immunocompromised hosts (Maillard and Centeleghe, 2023).

Phage-encoded depolymerases represent a class of specialized enzymes capable of degrading biofilm extracellular matrices, thereby sensitizing embedded bacteria to antimicrobial agents (Wang et al., 2024; Shahed-Al-Mahmud et al., 2021). These enzymes are phylogenetically classified into two groups: structural depolymerases, which constitute the majority of characterized variants and are genomically linked to phage structural proteins (Cheetham et al., 2024; Guo et al., 2023). Soluble depolymerases are typically encoded by discrete genetic modules that direct the production of secreted proteins (Pires et al., 2016). Functionally, these enzymes cleave bacterial surface polysaccharides, generating characteristic translucent halos around phage plaques, whose diameter correlates with enzymatic efficacy (Dunstan et al., 2021). Mechanistic studies demonstrate these proteins operate as either hydrolases or lyases based on their catalytic domains (Topka-Bielecka et al., 2021). Notably, many studies of depolymerases-antibiotics combinations therapy have demonstrated significantly improved efficacy against biofilm-associated infections compared with monotherapy (Islam et al., 2024).

Phage adsorption to host cells is primarily mediated by receptor-binding proteins (RBPs), which specifically recognize bacterial surface structures (Gao et al., 2023). This receptor recognition constitutes the first critical steps of phage infection and determines host range specificity (Farquharson et al., 2021). The selective binding to cognate receptors enables phages to identify and infect suitable hosts within complex bacterial communities (Dicks and Vermeulen, 2024). During the adsorption process, phage-encoded depolymerase facilitates infection by cleaving cell surface polysaccharides to expose underlying receptor molecules. Following receptor binding phage tail enzymes locally degrade peptidoglycan, inducing conformational changes that permit viral genome injection into the host cytoplasm (Letarov and Kulikov, 2017). Phage receptors are defined as specific bacterial surface structures recognized by RBPs and their availability directly governs infection efficiency. However, bacteria can evolve resistance through receptors modification, such as mutations that alter receptor structure and thereby block phage adsorption (Costa et al., 2024; Zhang et al., 2020; Eriksen et al., 2020).

In our previous study, *Klebsiella pneumoniae* LPKP (NCBI: KR824099) (hereinafter referred to as LPKP) was isolated from the lung of a musk deer (*Moschus berezovskii*) (Zhao et al., 2017). LPKP was detected as an ST29-type strain with relatively strong antibiotic resistance. Phage vB_KpnS_MK54 (NCBI: MW119258) was isolated from drinking water samples of *Moschus berezovskii* and its whole genome was sequenced (Lu et al., 2022).

In this study, potential LPS-associated factors involved in phage MK54 adsorption using SWISS-MODEL for protein structure prediction and GRAMM for protein-protein rigid docking, followed by experimental evaluation using gene deletion mutants and phage adsorption assays. Interestingly, phage MK54 formed plaques surrounded by halo zones *K. pneumoniae* on double-layer agar plates, suggesting the presence of a putative depolymerase associated with phage infection. Therefore, the putative depolymerase was expressed *in vitro* using the pET32a vector and its enzymatic activity and its potentiating effect in combination with antibiotics on LPKP biofilm were evaluated. These findings highlight the potential of Dep_Zy for controlling biofilms formed by.

## 2 Materials and methods

### 2.1 Bacteria, phage and growth conditions

*Klebsiella pneumoniae* LPKP and phage MK54 was isolated in our previous study (Lu et al., 2022). Detailed information on the engineered strains, vectors, and experimental materials, including the reagents used in this study, is presented in Supplementary Table 1. Phage titration and propagation of phages were performed using the double-layer agar plate method (Jo et al., 2024).

### 2.2 Construction of gene deletion mutants

The upstream and downstream homologous arms of *lpxB* and *msbA* were amplified by PCR using LPKP genomic DNA as template, while the drug resistance gene *Kan* (including its promoter) were amplified from plasmid pET-28a. Primers are listed in Table 1. The recombinant suicide plasmids pRE112-*Kan*-*lpxB* and pRE112-*Kan-msbA* was constructed and electroporated into the diaminopimelate (DAP) auxotrophic strain *E. coli* WM3064 λpir, enabling subsequent conjugal transfer to LPKP (Li et al., 2021). Subsequently, the LPKP ΔlpxB and LPKP ΔmsbA mutants were generated through two-step homolo (Xi et al., 2019). The LPKP△lpxB and LPKP△msbA mutants were confirmed by PCR and DNA sequencing.

**TABLE 1:** Gene and primers information.

Genes	Sequence (5′–3′)	Tm (°C)	Size of sequence
*Khe*	F:ATGAAACGACCTGATTGCATTCGC R:TTACTTTTTCCGCGGCTTACCGTC	50	489bp
*pRE112*	F:AAGGCAGTTATTGGTGCC R:GCCGATATCCTATTGGCA	50	n+200bp
*M13*	F: CGCCAGGGTTTTCCCAGTCACGAC R: AGCGGATAACAATTTCACACAGGA	50	n+200bp
*lpxB*	P1:gc***TCTAGA***TCATTGTTTTGCTAACTCCAGTACG P2: ACGCGTTTTCTATGATCGTGCATATGCTGGAC P3: CACGATCATAGAAAACGCGTTTTTGTCG P4: c***GAGCTC***TGGCTGAACAGCGTCCC	64 67	420bp 419bp
*msbA*	P1:gc***TCTAGA***ATGCAGAACGATAAAGATCTCT P2: CGTTTTGTGACCCTTCGCGGACCA P3: CCGCGAAGGGTCACAAAACGTGCATCTGTTCA P4: c***GAGCTC***TCATTCGCCGAATTGCATCT	59 67	470bp 501bp
*msbA-orf*	F: GCATCGATTATCGGTCTG R: AACCAGCGCCACCTGGTC	57	816bp
*lpxB-orf*	F: TCCGGCGCCGTCTCAGCTT R: AAAATCGGCAGATCCACC	60	350bp
*Kan*	P1:cg***ACGCGT***GAAGATCCTTTGATCTTTTCT P2:cc***CATATG***CAGGTGGCACTTTTCG	64	836bp
*T7/T7TER*	T7: TAATACGACTCACTATAGGG T7TER: TGCTAGTTATTGCTCAGCGG	50	n+200bp
*DEP_Zy*	F:cg***GAATTC***ATGGCTACTACCCCAACT R:cc***AAGCTT***AACAGCTGTGCCAGT	55	2098bp

Restriction sites are indicated in italics and bold. Restriction enzyme protection bases are indicated in lowercase letters; the overlapping parts are indicated in underline form.

### 2.3 Evaluation of lPS-associated factors involved in phage vB_KpnS_MK54 adsorption

The double-layer agar plate method was used to evaluate the effects of lpxB and msbA deletion on phage vB_KpnS_MK54 propagation by comparing plaque formations between the mutants (△lpxB and △msbA) and wild-type strains.

### 2.4 Depolymerase Dep_Zy preparation and depolymerase spectrum analysis

The open reading frame (ORF) of the depolymerase gene Dep_Zy, excluding the stop codon, was cloned into the pET-32a expression vector using the EcoRI and HindIII restriction sites. The primers used for cloning are listed in Table 1. The recombinant plasmid was transformed into *E. coli* BL21 (DE3) cells for protein expression. Initial expression was performed with 1 mM IPTG at 37 °C for 4 h. The induction conditions were subsequently adjusted based on the solubility of the recombinant protein. His-tagged Dep_Zy was purified by the HyPur T Ni-NTA 6FF (His-Tag) PrePacked Gravity Column (Sangong Biotech, China) according to the manufacturer’s instructions. The purified protein was concentrated using a Ultrafiltration Centrifuge Tube 30KDa (Solarbio, Shanghai, China) and stored at −80 °C. Protein concentration was determined using a BCA Protein Assay Kit, and protein purity was verified by SDS-PAGE followed by Coomassie Brilliant Blue staining. Then, purified Dep_Zy protein was gradiently diluted in PBS. For activity assessment, 200 μL of log-phase bacterial strains (listed in Table 2) culture was spread on 0.6% LB agar plates to form bacterial lawns. After surface drying, 5 μL aliquots of each Dep_Zy dilution were spotted onto the lawns with PBS serving as negative control. Following overnight incubation at 37 °C, depolymerase activity was quantified by measuring the diameter of clearance zones (halos) formed on the bacterial lawns.

**TABLE 2:** Depolymerase spectrum of Dep_Zy.

Strain	Bacteria	Host	Depolymerase activity
LPKP	*Klebsiella pneumoniae*	Forest musk deer	+
KP18	*Klebsiella pneumoniae*	Cattle	−
BKP	*Klebsiella variicola*	Cattle	−
EC1	*Escherichia coli*	Cattle	−
EC2	*Escherichia coli*	Forest musk deer	−
EC3	*Escherichia coli*	Sheep	−
SC1	*Shigella Castellani*	Cattle	+
SC2	*Shigella Castellani*	Forest musk deer	−
SC3	*Shigella Castellani*	Sheep	−
SAL1	*Salmonella*	Pig	−
SAL2	*Salmonella*	Mouse	−
SAL3	*Salmonella*	Chicken	−
S.A1	*Staphylococcus aureus*	Forest musk deer	−
S.A2	*Staphylococcus aureus*	Cattle	−
S.AR	*Staphylococcus arlettae*	Cattle	−
S.S	*Staphylococcus sciuri*	Cattle	−

### 2.5 Depolymerization activity against LPKP

Biofilm degradation assay: 200 μL of logarithmic phase LPKP (1 × 10^6^ CFU/mL) was cultured in a 96-well plate at 37 °C for 48 h to establish mature biofilms. Following PBS washes (3×) to remove planktonic cells, gradiently diluted Dep_Zy (prepared in PBS) was added to test wells, with PBS-treated wells used as vehicle controls. After 2 h incubation at 37 °C, biofilm biomass was quantified using crystal violet staining, followed by solubilization and measurement of absorbance (595 nm) (Grossman et al., 2021). Three biological replicates were performed for each condition.

Biofilm formation inhibition assay: Logarithmic phase LPKP (1 × 10^6^ CFU/mL) was co-cultured with gradiently diluted Dep_ Zy (prepared in TSB medium) in a 96-well plate. The plates were cultured statically at 37 °C for 48 h to allow biofilm development. After incubation, the supernatant was carefully removed, and the wells were gently washed with PBS to eliminate non-adherent cells. Three biological replicates were performed for each condition. Biofilm biomass was quantified using crystal violet staining.

Calculation of biofilm inhibition and degradation: the percentages of biofilm inhibition and degradation were calculated based on the reduction in absorbance at 595 nm relative to the corresponding vehicle control wells.

### 2.6 Antibiofilm activity of depolymerase- antibiotic combinations

The experimental design included four treatment groups: PBS (control), Dep_Zy, antibiotics and the combination of antibiotics and depolymerase. Mature biofilms were grown in the 96-well plate and treated with 200 μL respective test reagents at 37 °C for 2 h, to evaluate their short-term antibiofilm activity and antibiotic-potentiating effects while minimizing potential confounding effects associated with prolonged incubation The concentrations of kanamycin and gentamicin used in the combination experiments were 50, 100, and 200 μg/mL (1×, 2×, and 4× MIC, respectively), while streptomycin was used at 100, 200, and 400 μg/mL (1×, 2×, and 4× MIC, respectively). After treatment, the wells were washed three times with sterile PBS to remove residual treatment solution. The remaining bacteria were then recovered by repeated pipetting with 200 μL sterile PBS, and viable bacterial counts were determined by the plate-counting method. Three biological replicates were performed for each treatment group.

### 2.7 Antibiofilm activity on biofilm-contaminated surgical surface model

Stainless-steel surfaces measuring 4 cm × 4 cm were used to simulate surgical table surfaces. Briefly, the logarithmic phase LPKP bacterial suspension was inoculated onto the test surface and incubated at 37 °C for 48 h to allow biofilm formation. The surfaces were then washed three times with sterile PBS. The experiment was divided into five groups: (1) control group, in which 3 mL PBS was added to each surface; (2) Dep_Zy group, in which 3 mL Dep_Zy (10 μg/mL) was added; (3) antibiotic group, in which 3 mL of antibiotics at the indicated concentrations was added; (4) combination immersion group, in which 3 mL of a mixture of Dep_Zy (10 μg/mL) and antibiotics at the indicated concentrations was added to immerse the surface; and (5) combination spray group, in which approximately 1.5 mL of the Dep_Zy-antibiotic mixture at the corresponding concentrations was evenly sprayed onto the surface using a spray bottle. All treatments were incubated at 37 °C for 2 h. After treatment, the surfaces were washed three times with sterile PBS to remove residual treatment solution. The remaining bacteria were then recovered by repeated pipetting with 1 mL sterile PBS, and viable bacterial counts were determined by the plate-counting method. Three biological replicates were performed for each treatment group.

### 2.8 Statistical analysis

All data were analyzed using Origin 2025 software package and expressed as the mean ± standard deviation (SD). Three biological replicates (*n* = 3) were performed for each experimental group. Statistical significance was determined by one-way analysis of variance (ANOVA) followed by Tukey’s multiple-comparison test. A *P*-value ≤ 0.05 was considered statistically significant.

## 3 Results

### 3.1 Construction of pRE112-based recombinant suicide plasmids

The homologous flanking regions of *lpxB* and *msbA*, along with the gene *Kan*, were successfully amplified and assembled through overlap extension PCR. Subsequent cloning yielded three recombinant suicide plasmids: pRE112-*Kan*, pRE112-*Kan-lpxB* and pRE112-*Kan-msbA*. DNA sequencing analysis confirmed the integrity and accuracy of all plasmid constructs (Figure 1).

**FIGURE 1:**
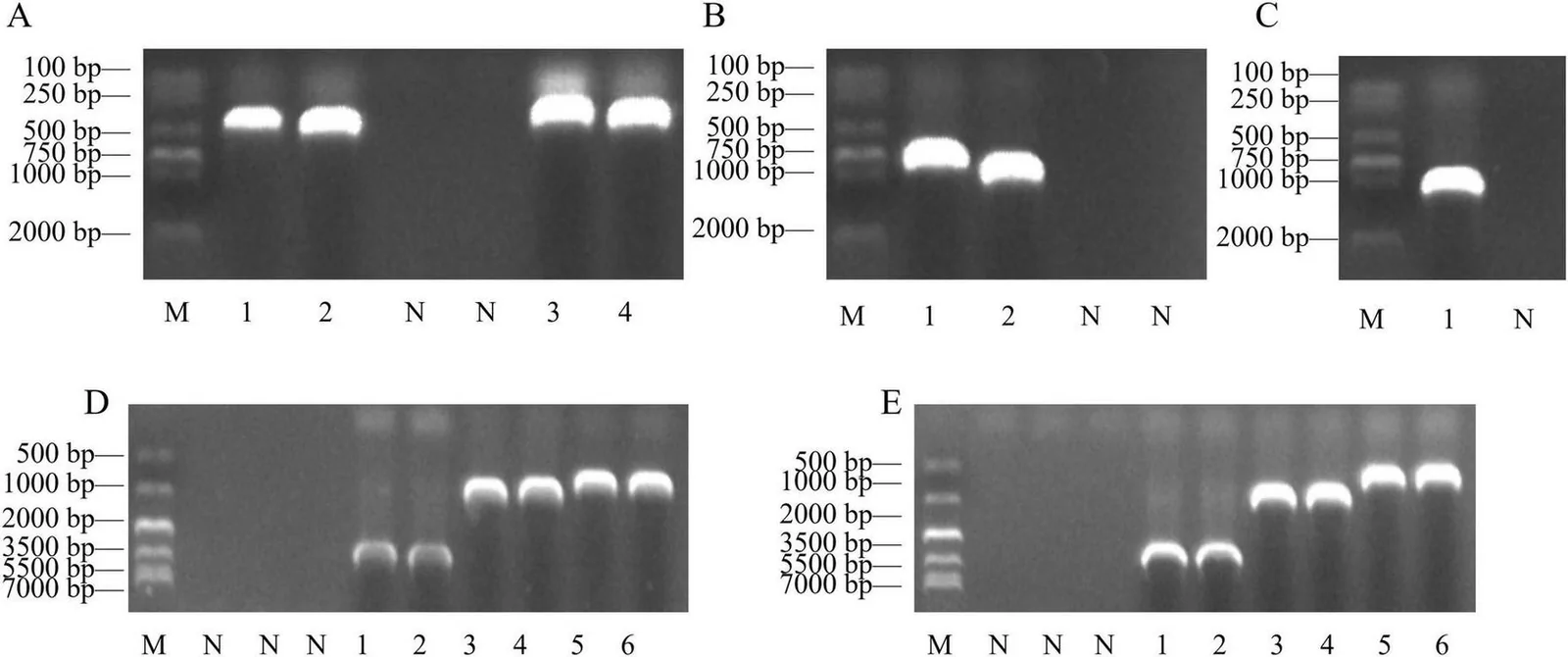
Construction of pRE112-basing recombinant suicide plasmids. **(A)** PCR amplification of the upstream and downstream of *msbA/lpxB*. M, DNA Marker D2000; 1–2, msbA-U and msbA-D; 3–4, lpxB-U and lpxB-D; N, negative control. **(B)** Overlap-extension of homology arms. 1, lpxB-UD; 2, msbA-UD. **(C)** Amplification of *Kan* gene. **(D)** Identification of pRE112(Kan)-△msbA recombinant plasmids by PCR. 1–2, PCR confirmation by pre112 primers; 3–4, PCR confirmation of gene Kan by Kan primers; 5–6, PCR confirmation of msbA-UD by msbA-P1/P4 primers. **(E)** Identification of pRE112(Kan)-△lpxB recombinant plasmids by PCR. 1–2, PCR confirmation by pRE112 primers; 3–4, PCR confirmation of gene Kan by Kan primers; 5–6, PCR confirmation of lpxB-UD by LpxB primers.

### 3.2 Construction of gene deletion mutant

The LPKP△lpxB and LPKP△msbA mutants were constructed by homologous recombination and confirmed by PCR (Figure 2). The *msbA* gene was deleted by 933-bp (residues 449–1265), resulting in the △msbA mutant lacking an internal region encoding amino acid residues 150–421. Similarly, the *lpxB* gene was deleted by 742-bp (residues 342–751), yielding the △lpxB mutant with a deletion of residues 114–376. Both mutants contained in-frame deletions within their respective open reading frames. The *msbA* and *lpxB* deletion mutants exhibited slower growth, rougher colony surfaces, smaller and more transparent colonies, and reduced culturability compared with the parental LPKP strain.

**FIGURE 2:**
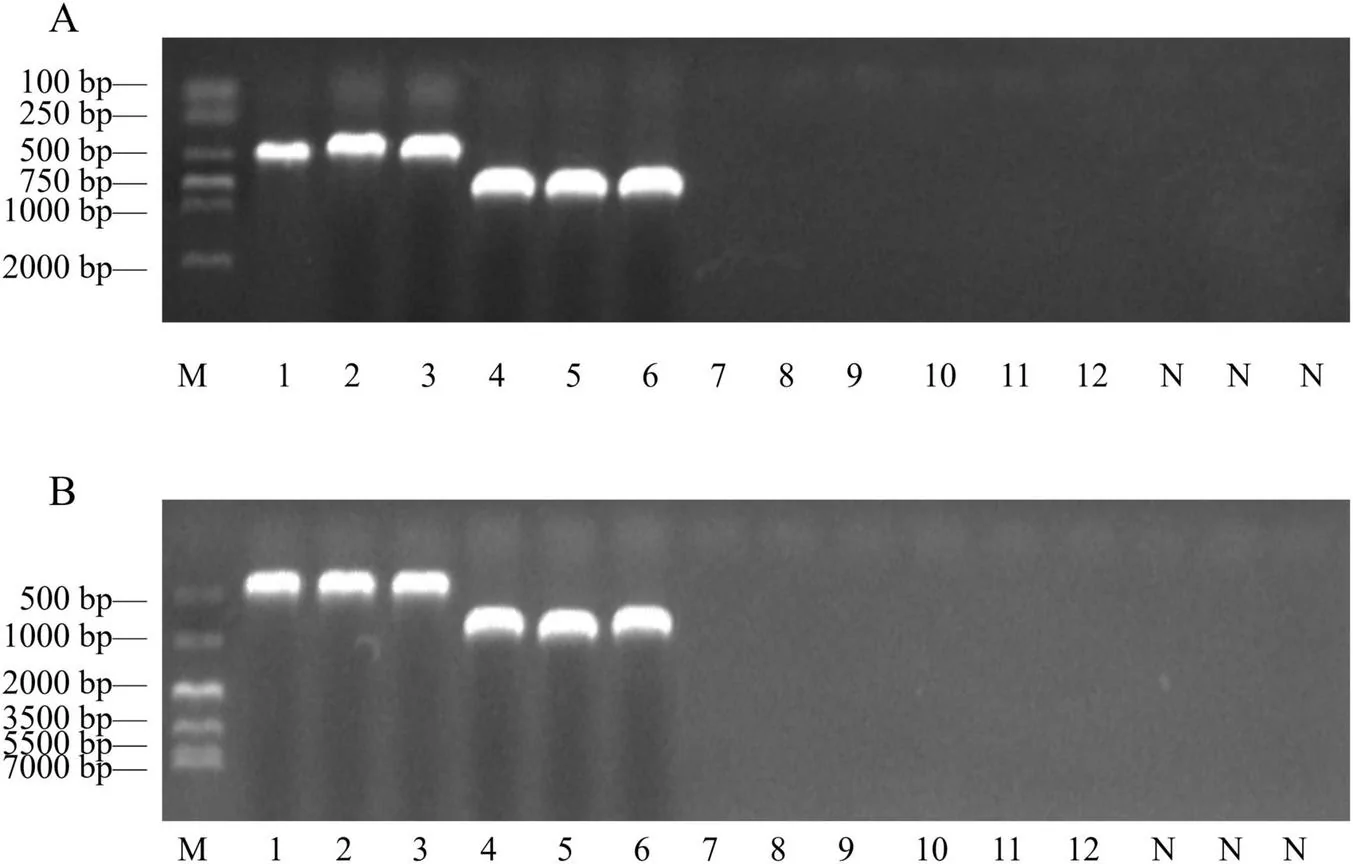
Construction of the △msbA and △lpxB mutant. **(A)** Identification of △msbA mutant by PCR. M, DNA Marker D2000; 1–3, PCR confirmation of LPKP by Khe primers; 4–6, PCR confirmation of msbA-UD by msbA-P1/P4 primers; 7–9, PCR confirmation by pre112 primers; 10–12, PCR confirmation of △msbA by msbA primers; N, negative controls for each primer. **(B)** Identification of △lpxB mutant by PCR. 1–3, PCR confirmation of LPKP by Khe primers; 4–6, PCR confirmation of lpxB-UD by lpxB-P1/P4 primers; 7–9, PCR confirmation by pre112 primers; 10–12, PCR confirmation of △lpxB by lpxB primers.

### 3.3 Effects of lPS-associated factors involved in phage vB_KpnS_MK54 adsorption

The double-layer agar plate assay showed that phage MK54 plaque formation on the LPKPΔmsbA and LPKPΔlpxB mutants was significantly reduced compared with that to the wild-type strain, from 10^10^ PFU/mL to 8.83 × 10^7^ PFU/mL and 5.5 × 10^6^ PFU/mL, respectively (Figure 3). Since msbA and lpxB are involved in LPS transport and biosynthesis, respectively, these results suggest that LPS plays an important role in phage MK54 adsorption (Bohl et al., 2018; Bonifer and Glaubitz, 2021).

**FIGURE 3:**
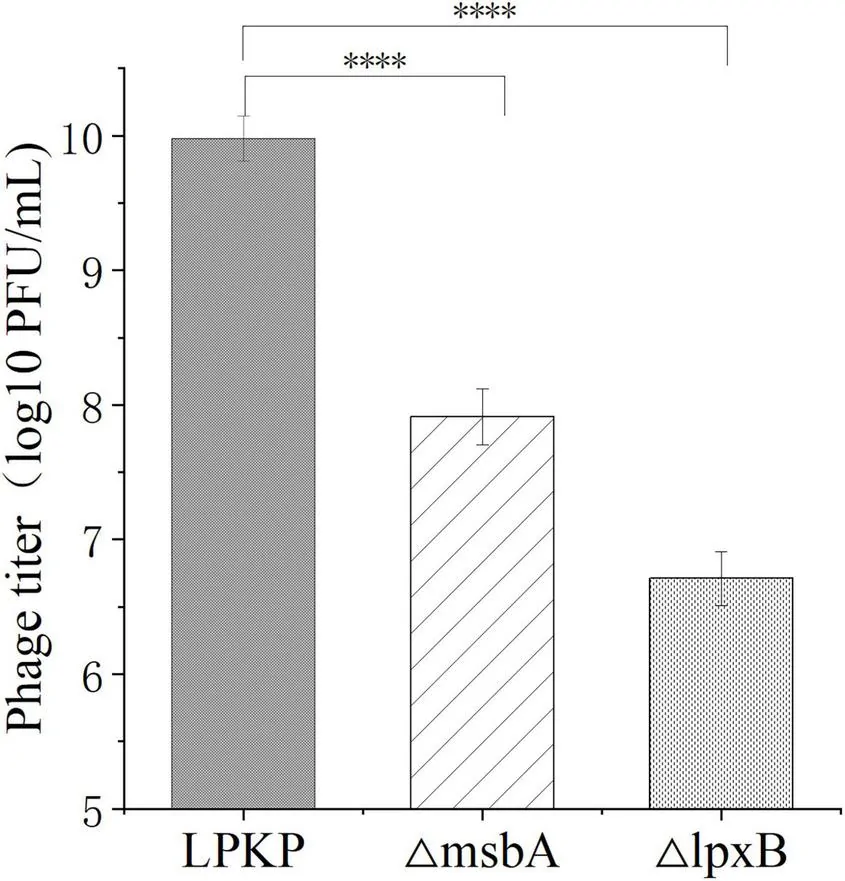
Effects of LPS-associated factors involved in phage vB_KpnS_MK54 adsorption. The wild strain LPKP, △lpxB mutant and △msbA mutant were cultured to the logarithmic phase and mixed with phage MK54, then the double-layer plate method was used to compare the difference in the number of phage plaques between the two mutants and the wild strain. Significance of difference was analyzed by one-way ANOVA (****p < 0.0001). Data are presented as mean ± SD (*n* = 3).

### 3.4 Cloning, expression and purification of the depolymerase

The Dep_Zy gene was successfully amplified and the recombinant expression plasmid pET32-Dep_Zy was constructed (Figure 4). The pET32-Dep_Zy was verified by PCR and DNA sequencing confirming the correct codon sequences. Subsequently, Dep_Zy protein was expressed and purified. SDS-PAGE and Western blot analysis confirmed the expression of a soluble recombinant Dep_Zy protein with an apparent molecular weight of approximately 95 kDa, corresponding to the Dep_Zy protein fused with the expression vector-derived tag. Although 0.5 mM IPTG induction at 37 °C for 4 h produced the highest overall expression level, a considerable fraction of the recombinant protein was present in the insoluble fraction. Therefore, lower-temperature (16 °C) induction with an extended induction period was subsequently employed to increase the proportion of soluble Dep_Zy and facilitate protein purification. The purified protein was diluted to 10 mg/mL using PBS buffer.

**FIGURE 4:**
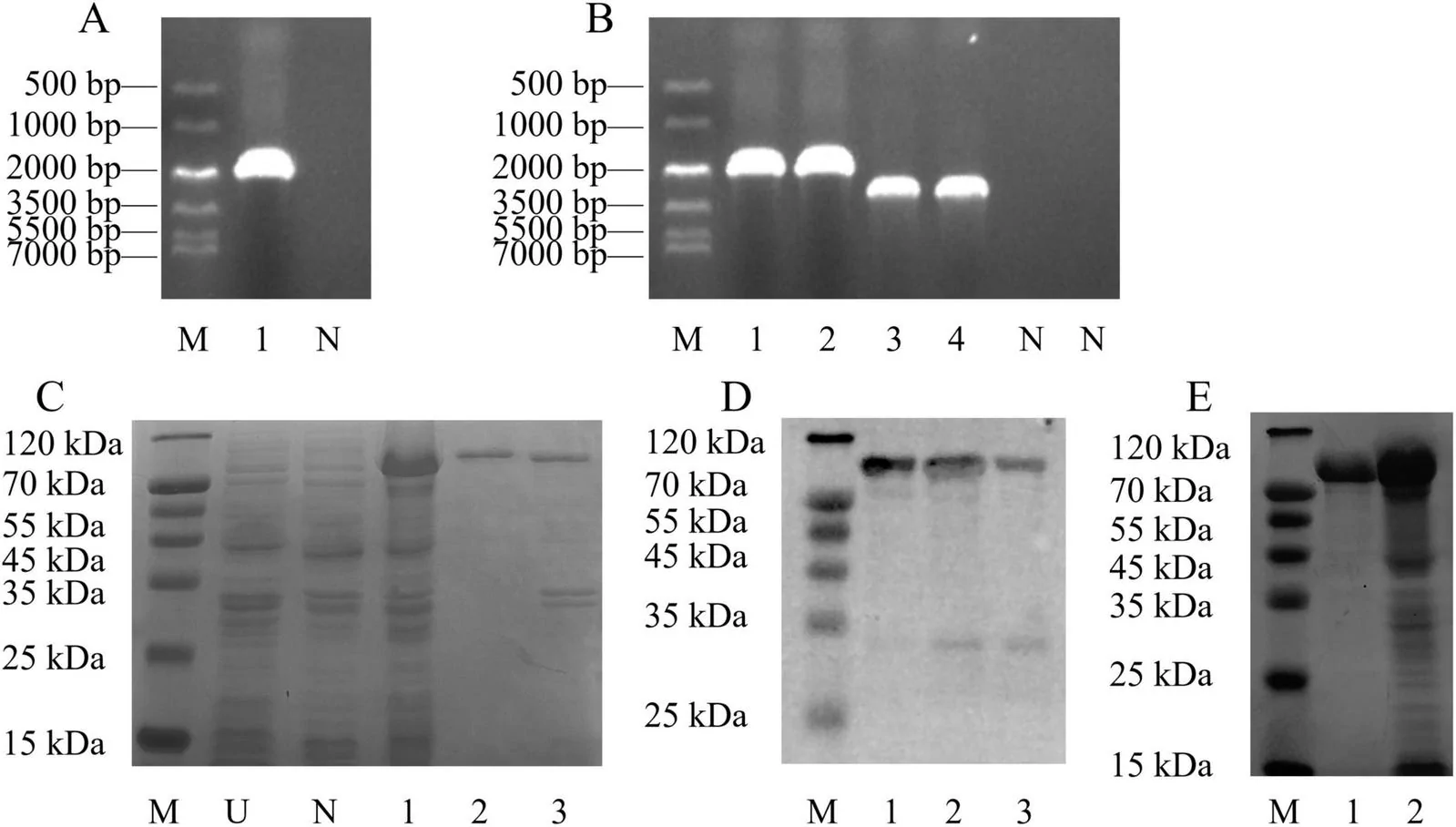
Cloning, expression and purification of the depolymerase. **(A)** Amplification of Dep_Zy by PCR. **(B)** PCR verification of the recombinant vector pET-32a-Dep. M, DNA Marker IV; 1–2, PCR confirmation by Dep primers; 3–4, PCR confirmation by T7/T7TER primers; N, negative controls. **(C)** Solubility analysis of Dep_Zy protein. M, protein marker (15–120 kDa); U, uninduced recombinant bacteria; N, BL21-pET-32a as negative control; 1–3, unbroken, supernatant and precipitate of the induced recombinant bacteria. **(D)** Western blotting of Dep_Zy. 1–3, precipitate, supernatant and unbroken of the induced recombinant bacteria. **(E)** Purification of Dep_Zy. 1, before purification; 2, after purification.

### 3.5 Activity spectrum and depolymerization activity of Dep_Zy

As shown in Figure 5: the depolymerase Dep_Zy exhibited strong depolymerase activity against *K. pneumoniae* LPKP, producing an inhibition zone of approximately 5 mm even at the low concentration of 0.1 ug/mL. Notably, Dep_Zy also exhibited depolymerase activity against a bovine-derived *Shigella*, although the substrate range of Dep_Zy was not systematically characterized in this study.

**FIGURE 5:**
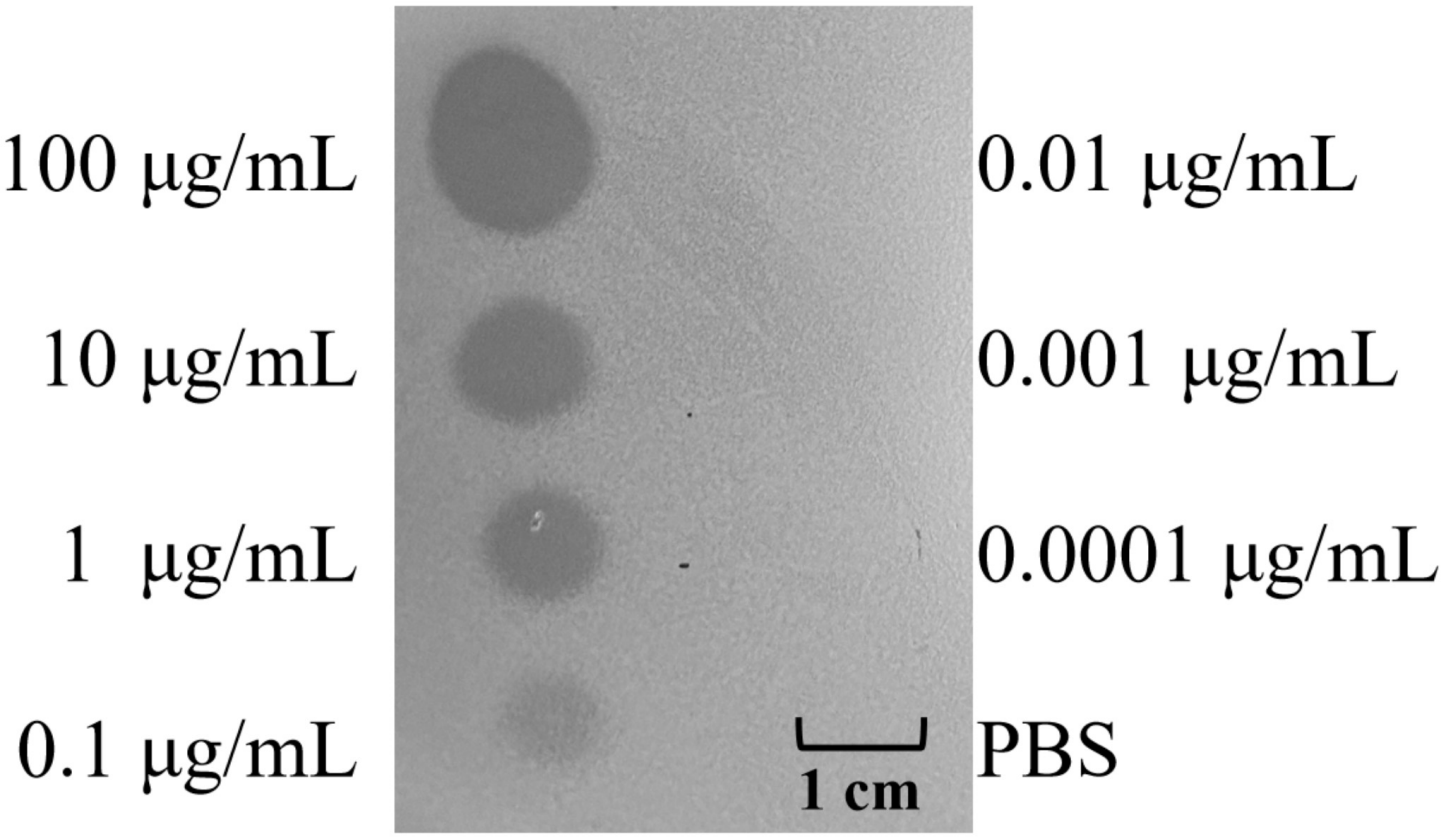
Depolymerase activity of the Dep_Zy against LPKP. Using the double-layer plate method, drop 5 μL of 0.001–100 μg/mL depolymerase onto a plate containing LPKP, respectively. PBS bufer was used as the control.

### 3.6 Depolymerization activity against LPKP biofilm

As shown in Figure 6, Dep_Zy (≥0.1 μg/mL) significantly inhibited LPKP biofilm formation (*p* < 0.01). Furthermore, after a 2-h incubation with mature LPKP biofilms, Dep_Zy at concentrations of ≥1 μg/mL significantly reduced the remaining biofilm biomass (*p* < 0.01). These results demonstrate that Dep_Zy exhibits anti-biofilm activity, effectively preventing biofilm formation and disrupting established biofilms.

**FIGURE 6:**
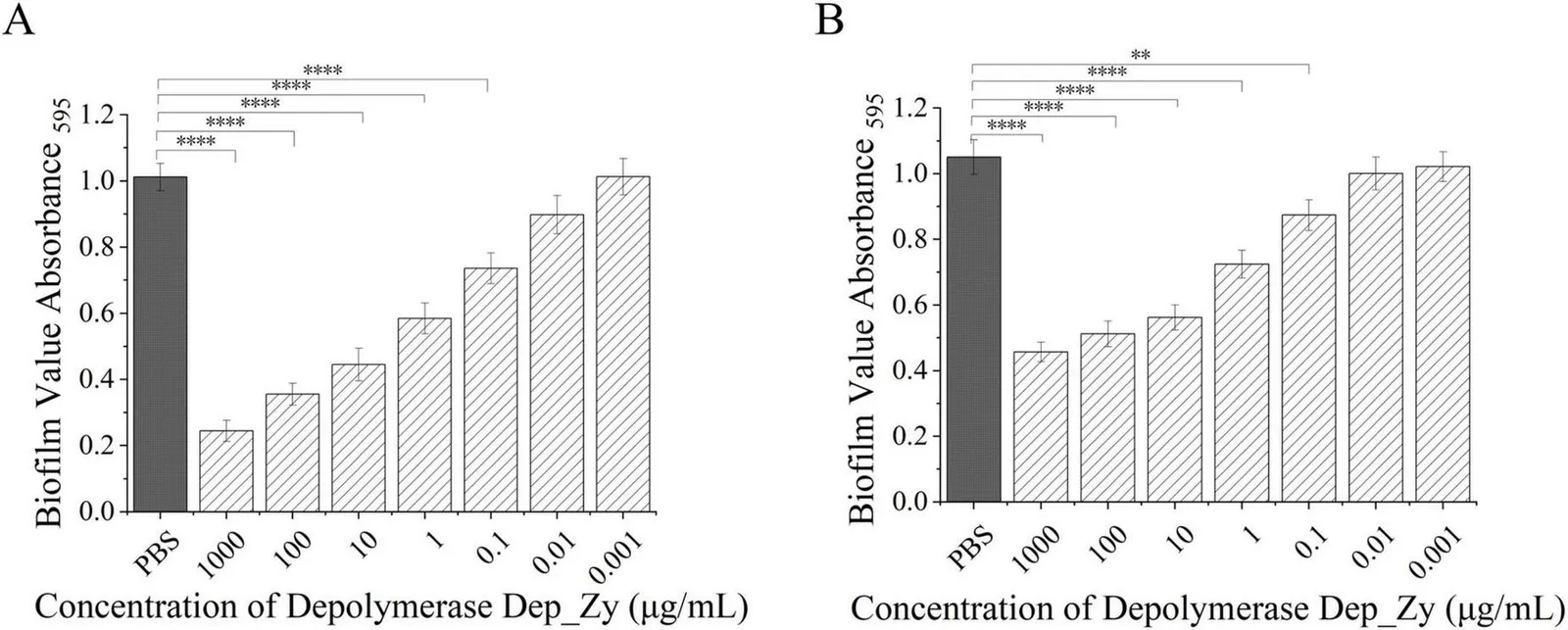
Antibiofilm activity of depolymerase Dep_Zy. **(A)** Dep_Zy inhibits biofilm formation. Dep_Zy was incubated with LPKP in 96-well plates for 48 h. **(B)** Dep_Zy degrades formed biofilm. After the LPKP biofilm matured in 96-well plates, Dep_Zy was added and incubated for 2 h. The biofilm was semi-quantitatively measured by crystal violet staining and the absorbance was measured at 595 nm. PBS buffer was used as negative control. Significance of difference was analyzed by one-way ANOVA (**p < 0.01, ****p < 0.0001). Data are presented as mean ± SD (*n* = 3).

### 3.7 Antibiofilm activity combined with antibiotics

Depolymerases can degrade bacterial surface polysaccharides but generally lack direct bactericidal activity. To enhance their antimicrobial efficacy, this study investigated the potentiating effect of combining depolymerase Dep_Zy with antibiotics (Figure 7). Dep_Zy (100 μg/mL) alone exhibited no bactericidal activity against biofilm-embedded bacteria. Treatment with kanamycin or gentamicin at 50 μg/mL, or streptomycin at 100 μg/mL, individually resulted in no significant reduction in bacterial counts compared with untreated biofilms (*P* > 0.05). However, combining Dep_Zy with these antibiotics significantly enhanced their antimicrobial efficacy, as evidenced by a marked reduction in viable bacterial counts in biofilms. These results demonstrate that Dep_Zy enhances the susceptibility of biofilm-associated bacteria to antibiotics, thereby potentiating antibiotic activity.

**FIGURE 7:**
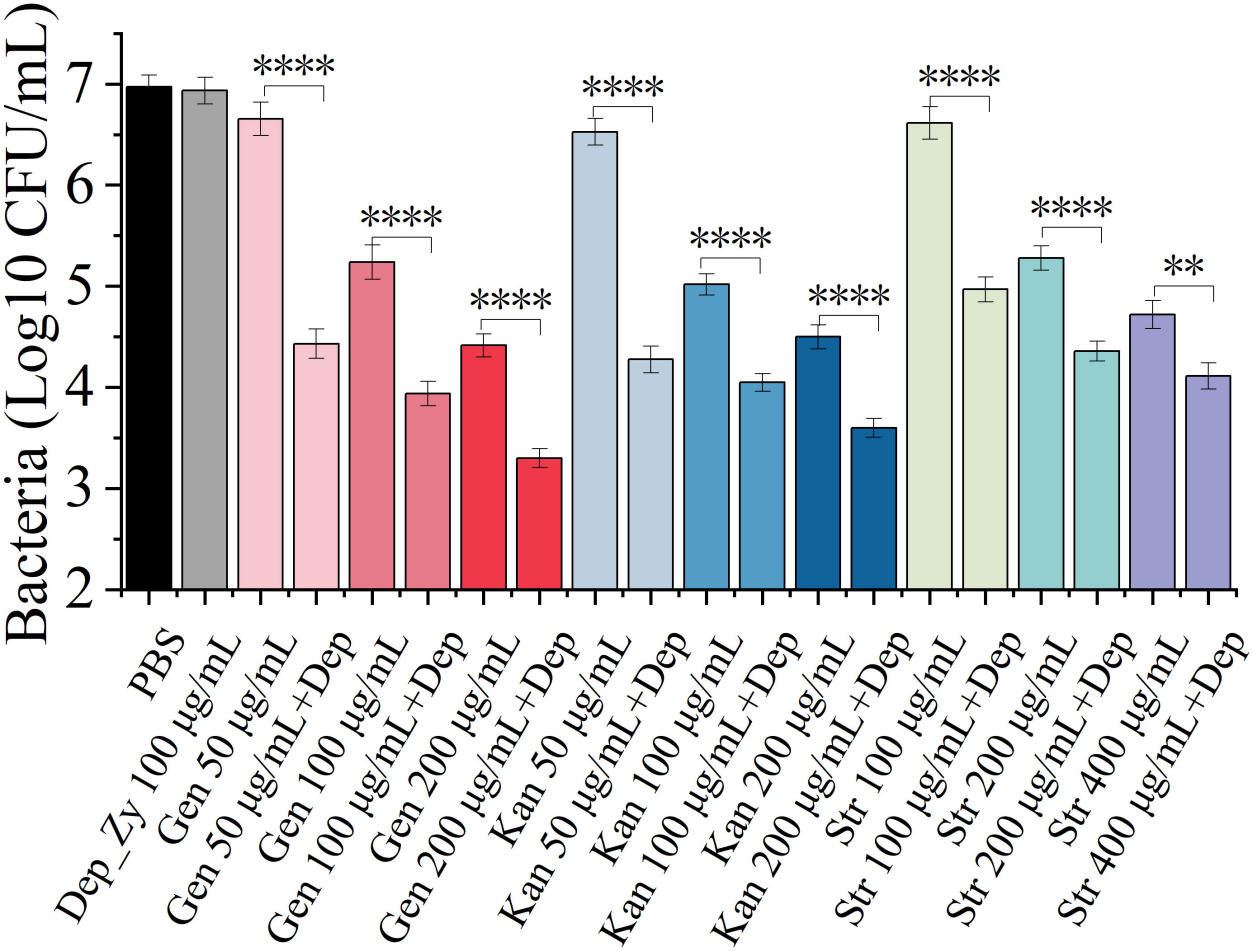
Antibacterial activity of depolymerase Dep_Zy combined with antibiotics. After the LPKP biofilm matured in 96-well plates, wash it three times with PBS. Then, Dep_Zy, antibiotics and their combination were added to the wells, respectively, and incubated for 2 h. After incubation, wash each well three times with PBS, followed by colony counting. Gen, gentamicin; Kan, kanamycin; Str, streptomycin. Significant differences are indicated by one-way ANOVA (**P < 0.01, ****P < 0.0001). Data are presented as mean ± SD (*n* = 3).

### 3.8 Antibiofilm activity on biofilm-contaminated surgical surface model

As shown in Figure 8, in the biofilm-contaminated surgical table model, the combined treatment of Dep_Zy with either gentamicin, kanamycin, or streptomycin enhanced the bactericidal efficacy against biofilm-embedded bacteria, achieving a 1–2 log reduction in bacterial counts compared to antibiotics treatment alone. This potentiating effect was observed with both immersion and spray disinfection methods with no significant difference in efficacy between the two disinfection approaches.

**FIGURE 8:**
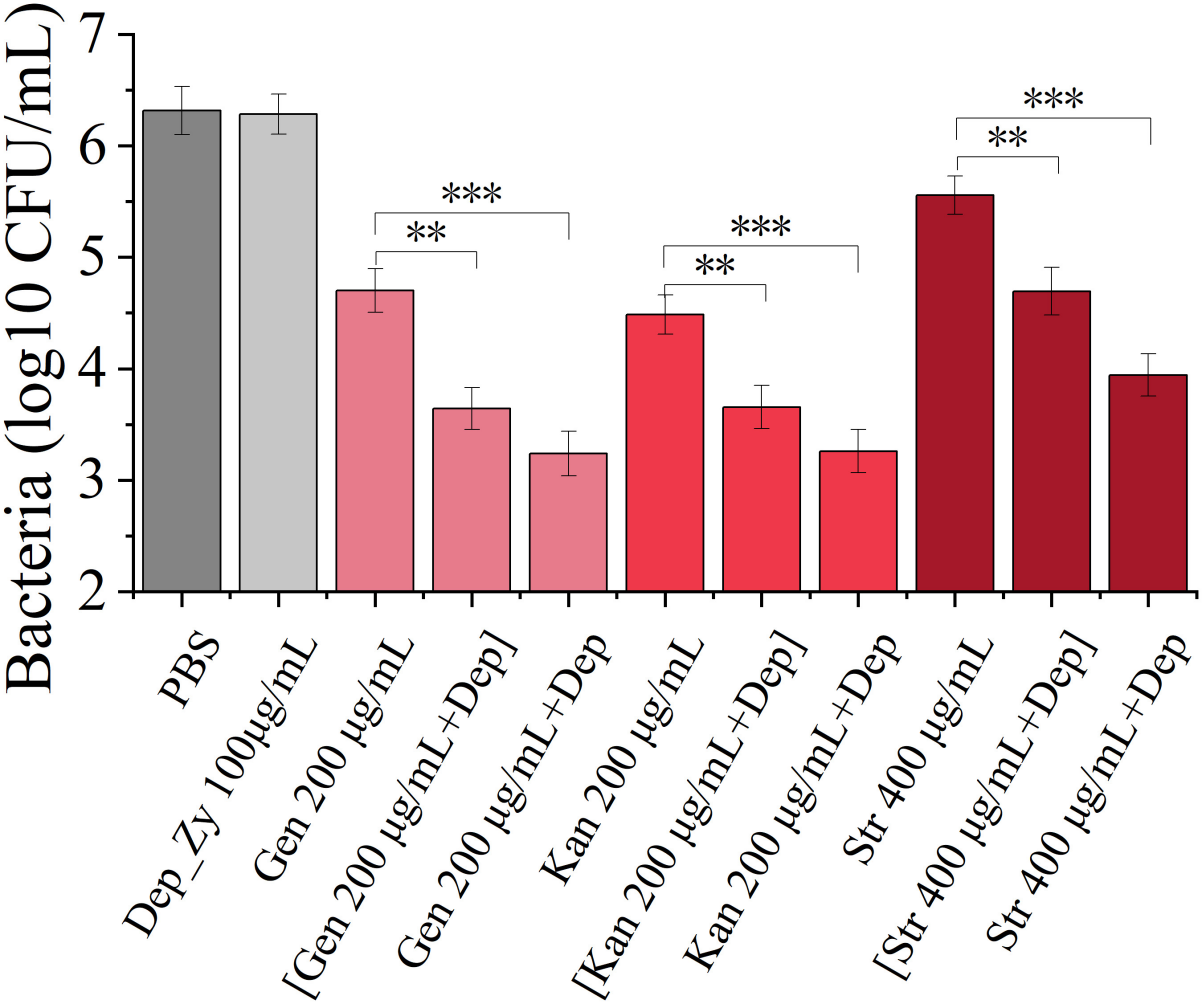
Antibacterial activity of Dep_Zy on biofilm-contaminated operating table model. Using stainless-steel trays to simulate the surface of an operating table and construct a biofilm-contaminated operating table model. Disinfection was performed using depolymerase and antibiotics in both spraying and soaking methods, and the disinfection effect was evaluated based on viable bacteria count. Gen, gentamicin; Kan, kanamycin; Str, streptomycin. [Gen/Kan/Str] indicates spray group. Significant differences are indicated by one-way ANOVA (**P < 0.01, ***P < 0.001). Data are presented as mean ± SD (*n* = 3).

## 4 Discussion

In this study, we found that the antibiotic resistance of *K. pneumoniae* LPKP rendered the chloramphenicol resistance marker carried by the suicide plasmid pRE112 ineffective, thereby hindering gene knockout via the suicide plasmid method. In response to this problem, we introduced an additional kanamycin resistance marker into the plasmid and constructed a new plasmid. This modification enabled the successful generation of the mutants LPKPΔlpxB and LPKPΔmsbA through double homologous recombination. Subsequently, we found that deletion of *msbA* or *lpxB* markedly reduced phage MK54 propagation on LPKP, suggesting that LPS-associated pathways play an important role in phage adsorption.

The genes *msbA* and *lpxB*, which are involved in LPS transport and biosynthesis, respectively, were found to significantly affect phage adsorption. The *msbA* encodes a transporter that facilitates the translocation of lipid A across the inner membrane (Bohl et al., 2018), while *lpxB* encodes a lipid-A-disaccharide synthase responsible for lipid A precursor formation, anchoring LPS to the outer membrane (Bonifer and Glaubitz, 2021; Maldonado et al., 2016). The *msbA* and *lpxB* deletion mutants exhibited notable phenotypic changes compared with the parental LPKP strain, as described above, suggesting that disruption of these genes may affect bacterial growth and cell-envelope-related properties. Furthermore, deletion of *msbA* or *lpxB*, which are involved in LPS transport and biosynthesis, respectively, markedly reduced phage MK54 adsorption, potentially due to alterations in the abundance, structure, or surface presentation of LPS. These findings further indicate LPS likely contributes to phage MK54 adsorption to LPKP. In addition, based on our previous bioinformatic analysis, molecular docking predicted favorable interactions between the receptor-binding proteins of phage MK54 and MsbA or LpxB, with relatively low binding free energies. However, considering that MsbA and LpxB are involved in LPS transport and biosynthesis, respectively, and are not directly exposed on the bacterial surface, these docking results should be interpreted with caution and do not constitute direct evidence of receptor recognition. Instead, the observed interactions may support the potential involvement of LPS-associated pathways in phage adsorption, while the reduced adsorption observed in the corresponding deletion mutants may result from alterations in LPS abundance, structure, or surface presentation. The precise LPS structure(s) recognized by MK54 remain to be determined. LPS is the most widely reported receptor for Gram-negative phages (Choi et al., 2017). Consistent with our findings, previous studies have shown LPS significantly influence phage adsorption. For instance, Ding identified lipopolysaccharides (LPSs) on the surface of the host *M. aerodenitrificans* strain LH9 as host receptors involved in the adsorption of the phage phiNY (Ding et al., 2024). Gong et al. (2021) demonstrated that outer membrane protein and LPS mediate phage Psm140 adsorption, while Filippov et al. (2011) further confirmed that phages utilize multiple core LPS regions as receptors in *Yersinia pestis*.

Notably, even with *msbA* and *lpxB* deleted, a small number of phage MK54 particles still managed to adsorb to LPKP. This implies that additional receptor structures may be involved, such as capsular polysaccharides or outer membrane proteins. Further experimental validation is necessary to elucidate the full receptor spectrum of phage MK54, which will help comprehensively understand the complexity of phage-host interactions.

In addition to exploring the receptor, we also investigated the anti-biofilm activity of the depolymerase Dep_Zy from phage MK54. Dep_Zy is a tail fiber protein of phage MK54, and SDS-PAGE analysis showed that the purified protein was soluble and had a molecular weight of approximately 95 kDa. Although 37 °C was identified as the optimal induction temperature for Dep_Zy expression, substantial inclusion body formation was observed at this temperature, likely due to the limited folding capacity of the expression host. Lowering the induction temperature to 16 °C and prolonging the induction period resulted in a higher yield of soluble Dep_Zy with improved purity. Functionally, Dep_Zy exhibited potent depolymerase activity against *K. pneumoniae* LPKP. As little as 0.1 μg/mL of purified Dep_Zy was sufficient to exhibit detectable depolymerase activity. Furthermore, Dep_Zy significantly inhibited biofilm formation and degraded mature biofilms in a concentration-dependent manner. At 10 μg/mL, Dep_Zy inhibited approximately 55% of biofilm formation, increasing to approximately 80% at 1,000 μg/mL. For mature biofilms, 1 μg/mL Dep_Zy resulted in approximately 30% degradation, whereas increasing the concentration to 1,000 μg/mL increased the degradation rate to approximately 60%. These findings suggest that Dep_Zy is more effective at preventing biofilm formation than at degrading preformed biofilms, highlighting its potential as a preventive antibiofilm agent.

Given the multidrug-resistant nature of LPKP, it is noteworthy that Dep_Zy significantly enhanced the susceptibility of biofilm-associated bacteria to antibiotics. When combined with three aminoglycoside antibiotics (kanamycin, gentamicin, and streptomycin), Dep_Zy potentiated the antibacterial effects of antibiotics, reducing viable cell counts by 1–3 log within 2 h. This enhanced activity may be related to complementary mechanisms between polysaccharide degradation by Dep_Zy and antibiotic-mediated bacterial killing. The extracellular polymeric matrix provides structural protection to biofilm-associated bacteria and can limit the accessibility of antimicrobial agents. Therefore, degradation of polysaccharide components by Dep_Zy may weaken the biofilm matrix and potentially facilitate antibiotic access to bacterial cells. Such matrix-mediated enhancement has also been discussed in recent studies of phage-antibiotic combinations. For example, Tchatchiashvili et al. (2026) reported that phage treatment degraded α- and β-polysaccharides within the extracellular polymeric substance of MDR *Klebsiella quasipneumoniae* biofilms and suggested that matrix degradation may contribute to improved antibiotic penetration and enhanced bacterial clearance. These findings support the possibility that disruption of the biofilm matrix by phage-derived enzymes may complement the activity of conventional antibiotics.

The observed potentiation may also be influenced by the physiological effects of antibiotic treatment on bacterial cells. Antibiotic-induced stress, alterations in membrane permeability or cell-envelope integrity, and reduced bacterial fitness have been proposed as factors that can influence bacterial susceptibility to phage- or enzyme-mediated antibacterial activity. However, the specific contribution of increased antibiotic penetration, changes in bacterial physiology, or other mechanisms was not directly investigated in the present study and requires further experimental validation.

The interaction between phages and antibiotics has attracted increasing attention as a potential strategy for improving bacterial and biofilm control. Recent studies have demonstrated that the outcome of phage-antibiotic combinations can depend on the mechanisms of action and relative concentrations of the two agents, highlighting the complexity of these interactions (Tchatchiashvili et al., 2026). In the present study, we did not quantitatively establish classical phage-antibiotic synergy, but rather observed that Dep_Zy, a phage-derived enzyme, potentiated the antibacterial effects of the tested aminoglycosides against LPKP biofilms. Therefore, these findings should be interpreted as antibiotic potentiation rather than classical phage-antibiotic synergy.

To simulate an operating table surface, stainless-steel trays were used to establish a biofilm contamination model. The antibiofilm effects of Dep_Zy described above were consistently observed in this model. Moreover, effective biofilm removal was achieved through spray application of Dep_Zy, highlighting its potential for practical application. The present study evaluated the short-term antibiofilm effects of Dep_Zy and its combination with antibiotics using a 2-h treatment period. Longer treatment durations may provide additional information regarding the time-dependent activity of Dep_Zy and its antibiotic-potentiating effects and should be investigated in future studies.

Our results are consistent with several previous reports. The depolymerase from phage 168 degraded 45% of *K. pneumoniae* biofilm and enhanced serum bactericidal activity by 2 logs (Sun et al., 2023). Bansal et al. (2015) reported that 10 μg/mL of depolymerase inhibited 56% of biofilm formation and enhanced the bactericidal activity of gentamicin, resulting in a 3-log reduction in viable bacterial counts. Dpo10 was reported to inhibit 90% of biofilm formation and reduce viable bacterial counts on steel surfaces by 2.56 logs when used in combination with antibiotics (Park and Park, 2021). Taken together, these findings indicate that phage-derived depolymerases may complement conventional antibiotics by weakening biofilm-associated protective barriers. In the present study, Dep_Zy showed substantial antibiofilm activity and potentiated the antibacterial effects of aminoglycosides against LPKP biofilms, supporting its potential for further investigation as an adjunctive antibiofilm agent.

With the increasing prevalence of antibiotic resistance, bacteriophage-derived depolymerases have emerged as promising antibiofilm agents. In this study, Dep_Zy effectively disrupted *K. pneumoniae* LPKP biofilms and enhanced the bactericidal activity of antibiotics, highlighting its potential as an antibiofilm agent and adjunctive strategy for controlling multidrug-resistant *K. pneumoniae* infections.

## 5 Conclusion

Compared with the wild-type strain, phage MK54 propagation on the LPKP△lpxB and LPKP△msbA mutants was significantly reduced, suggesting that LPS-associated pathways play an important role in phage MK54 adsorption.

The depolymerase Dep_Zy of phage MK54 exhibited depolymerase activity against *Klebsiella pneumoniae* and showed strong antibiofilm activity. Moreover, Dep_Zy enhanced the susceptibility of biofilm-associated bacteria to antibiotics when used in combination with antibiotics.

## Data Availability

The datasets presented in this study can be found in online repositories. The names of the repository/repositories and accession number(s) can be found in the article/Supplementary material.
