# Supplementary material for: Phage vB_KpnS_MK54: lPS-associated factors involved in phage adsorption and depolymerase-driven anti-biofilm effects
Source: Front Microbiol. 2026 Oct 9;17:1963357. doi: 10.3389/fmicb.2026.1963357 (PMC13690813; doi:10.3389/fmicb.2026.1963357)
Supplement: Supplementary file 1 [file Table_1.docx]

Supplementary Material

# 1 Supplementary methods

2.1&2.4 Double-layer agar plate method:The double-layer agar plate method was used to determine the phage titer of MK54 and to quantify plaque formation on LPKP and its derivative strains. Briefly, an overnight culture of the bacterial host was diluted in fresh medium and grown to the exponential phase. The bacterial culture was then mixed with an appropriate dilution of the phage suspension. The mixture was combined with molten soft agar maintained at approximately 45–50°C and immediately poured onto the surface of pre-solidified nutrient agar plates. After the soft agar layer had solidified, the plates were incubated at 37 °C under the conditions specified below. Following incubation, well-isolated plaques were counted on plates containing an appropriate number of plaques. Phage titers were calculated as plaque-forming units per milliliter (PFU/mL) according to the following relationship:

$$Phagetiter(PFU/mL)=numberofplaques\times dilutionfactor/volumeofphagesuspensionplated(mL).$$

For each phage sample, plates from appropriate dilution levels were used for enumeration, and the phage titer was calculated from plates with countable and well-separated plaques.

2.2 Construction of gene deletion mutants：The upstream and downstream homologous arms of lpxB and msbA were amplified by PCR using LPKP genomic DNA as template, while the drug resistance gene Kan (including its promoter) was amplified from plasmid pET-28a. Primers are listed in Table 1. Through overlap extension PCR, the homologous arms were fused with the Kan cassette to generate three hybrid fragments:△lpxB-Kan, △msbA-Kan and Kan alone. These fragments were first cloned into pMD19-T vector and transformed into E. coli DH5a competent cells for amplification. Subsequently, the Kan cassette was inserted into the suicide vector pRE112 via the XbaI/SacI restriction sites, generating pRE112-Kan. The homologous arms were then introduced into the pRE112-Kan using NdeI/MluI restriction sites, creating the recombinant plasmids pRE112-Kan-lpxB and pRE112-Kan-msbA. All constructs were initially transformed into E. coli SM10 λpir for plasmid propagation. Finally, The plasmids were extracted and electroporated into the diaminopimelate (DAP) auxotrophic strain E. coli WM3064 λpir, enabling subsequent conjugal transfer to LPKP. The recipient strain LPKP and donor E. coli WM3064 λpir strain were cultured overnight at 37 ℃ .Both strains were then diluted 1:100 into fresh medium and grown to mid-log phase (OD600≈0.6). Cells were harvested by centrifugation (6,000 rpm/min, 4 ℃ for 5 min), washed, and resuspended in fresh LB medium. The donor and the recipient strains were mixed by brief vortexing at a ratio of 3:1 ratio(v/v) pelleted (6000 rpm/min, 4 ℃, 3 min) and resuspended in 100 μL LB medium.The cell mixture was spotted on a LB plates supplemented with 25 μg/mL DAP and incubated at 37 ℃ for 48 h for conjugation. Following conjugation, the cells were collected, washed with PBS, and plated on LB agar containing 35 μg/mLCm and 50 μg/mL Kan to screen for LPKP colonies within tegrated suicide plasmid (single-crossover recombinants). Positive colonies were verified by PCR. Single-crossover strains were then diluted overnight in LB broth (35 μg/mL Cm, 50 μg/mL Kan), and plated on LB agar with 10% sucrose for counterselection. After 12h incubation at 37 ℃, double-crossover recombinants (∆msbA and ∆lpxB mutants) were obtained. The LPKP△lpxB and LPKP△msbA mutants were confirmed by PCR and DNA sequencing.

2.5 Preparation of LPKP biofilms in 96-well plates：LPKP was cultured overnight in TSB at 37°C with shaking. The overnight culture was diluted 1:100 in fresh TSB, and the bacterial suspension was further adjusted to approximately 1 × 10⁶ CFU/mL. A volume of 200 μL of the bacterial suspension was transferred into each well of a sterile, flat-bottom 96-well polystyrene microplate. Wells containing sterile TSB without bacteria were included as negative controls. The microplate was incubated statically at 37°C for 48 h to allow biofilm formation.

After incubation, the culture medium containing planktonic cells was carefully aspirated from each well without disturbing the attached biofilm. Each well was gently washed three times with 200 μL sterile PBS to remove loosely attached and non-adherent cells. After the final wash, the plate was used immediately for subsequent biofilm treatment or analysis. Unless otherwise specified, biofilms prepared using this procedure were considered mature LPKP biofilms.

2.5 crystal violet staining: , 200 μL of 4% paraformaldehyde was added to each well to fix the biofilms for 15 min. The wells were then washed three times with PBS and air-dried. Subsequently, 200 μL of 1% crystal violet solution was added to each well and incubated for 15 min for staining. The wells were washed three times with PBS and air-dried again. Finally, 33% acetic acid was added to each well and incubated for 10 min to solubilize the bound crystal violet. The absorbance was measured at 595 nm to quantify the attached biofilm biomass.

# 2 Supplementary Figures and Tables

## 2.1 Supplementary table

**Table S1.** Strains, plasmids, and experimental materials used in this study.

| Strains, plasmids and reagents | Source |
| --- | --- |
| pRE112-Kan-lpxB(pRE112 containing lpxB homologous arms and Kan cassette)  pRE112-Kan-msbA(pRE112 containing msbA homologous arms and Kan cassette)  pET-32a-Dep_Zy（pET-32a carrying Dep_Zy ORF）  LPKP(Wild-type K. pneumoniae)  LPKPΔlpxB(In-frame deletion of lpxB)  LPKPΔmsbA(In-frame deletion of msbA) | This study |
| 2×Pfu Master Mix (kp201), | Tiangen, Beijing, China |
| 2×Rapid Taq Master Mix ([P222-01](https://bio.vazyme.com/product/127.html)), [FastPure Gel DNA Extraction Mini Kit](https://bio.vazyme.com/product/18.html) ([DC301-01](https://bio.vazyme.com/product/18.html)) | Vazyme, Nanjing, China |
| T4 ligase (2011A), pMD19-T (3271), QuickCut EcoR I (1611), QuickCut Hind III (1615)、Cut XbaI (1093A)、Cut SacI (1078A), TaKaRa BCA Protein Assay Kit(T9300A) | TAKARA, Beijing, China |
| D2000 DNA Marker(DM2150), DNA Marker IV (DM1005), 50×TAE Buffer (SL2100), Coomassie brilliant blue R250 Protein Stain Reagent (SL1290) | Coolaber, Beijing,China |
| LB broth(ST156), Agar(ST004E), BeyoColor™ Prestained Color Protein Marker (P0078), BeyoECL Plus9 (P0018S), QuickBlock™ Protein-Free Blocking Buffer for Western Blot (P0240), SDS-PAGE Sample Loading Buffer (P0015) | Beyotime, Shanghai, China |
| Plasmid Mini Extraction Kit (NB 102-200T) | Oriscience, Chengdu, China |
| Ampicillin, Sodium Salt(A8180), Chloramphenicol (C8050), Kanam-ycin (K8020), SolarRed Agarose LE (A8202), 0.01M PBS (P1010), Isopropyl β-D-Thiogalactopyranoside (I8070), 96-Well Cell Culture Plate (YA0585), Tris-Tricine-SDS-PAGE (P1320), Sodium Dodecylsulfate (S8010), Tris (Hydroxymethyl) Aminomethane (T8060), Glycine (G8200), Ultrafiltration Centrifugal Tube 30KDa(UFC903096) | Solarbio, Shanghai, China |
| Cell Culture Dish (715001) | NEST Biotechnology Co, Wuxi, China |
| pET-32a(+)DNA (B540186), HyPur T Ni-NTA 6FF (His-Tag) PrePacked Gravity Column (C600793), Binding/ Wash Buffer (C600326), Anti-6X His Tag mouse monoclonal antibody (D191001), HRP-conjugated Goat Anti-Mouse IgG (C650201) | Sangong biotech, Shanghai,China |
| Polyvinylidene Fluoride (PVDF-45-001) | Bltlux,Guangzhou, China |
| DH5α(EC007H-M), BL21 (DE3)(EE008-H-S) | Bio-Rabit, Shanghai China |
| E.coli SM10 with plasmid pRE112(ZT295) | Fenghuishengwu Changsha, China |
| E.coli WM3064(bio-116091) | Biobw, Beijing, China |
